# Application of self-anchored lateral lumbar interbody fusion in lumbar degenerative diseases

**DOI:** 10.1186/s12891-023-06974-x

**Published:** 2023-10-23

**Authors:** Kaihui Zhang, Haiwei Xu, Lilong Du, Yue Liu, Baoshan Xu

**Affiliations:** Department of Minimally Invasive Spine Surgery, Tianjin Hospital, 406 JieFangNan Road, Hexi District, Tianjin, 300211 People’s Republic of China

**Keywords:** Self-anchored cage, Degenerative disease, Spinal fusion, Surgical procedure, Lateral lumbar interbody fusion

## Abstract

**Study Design:**

This is a retrospective study.

**Objective:**

The aim of the study was to evaluate the efficacy of self-anchored lateral lumbar interbody fusion (SA-LLIF) in lumbar degenerative diseases.

**Methods:**

Forty-eight patients with lumbar degenerative disease between January 2019 and June 2020 were enrolled in this study. All patients complained of low back and leg pain, which were aggravated during standing activities and alleviated or disappeared during lying. After general anesthesia, the patient was placed in the right decubitus position. The anterior edge of the psoas major muscle was exposed through an oblique incision of approximately 6 cm, using an extraperitoneal approach. The psoas major muscle was then properly retracted dorsally to expose the disc. After discectomy, a suitable cage filled with autogenous bone graft from the ilium was implanted. Two anchoring plates were inserted separately into the caudal and cranial vertebral bodies to lock the cage. Clinical efficacy was evaluated using the visual analog scale (VAS) and Oswestry Disability Index (ODI). Lumbar lordosis, intervertebral disc height, spondylolisthesis rate, cage subsidence and fusion rate were also recorded.

**Results:**

A total of 48 patients were enrolled in this study, including 20 males and 28 females, aged 61.4 ± 7.3 (range 49–78) years old. Surgery was successfully performed in all patients. Lumbar stenosis and instability were observed in 22 cases, disc degenerative disease in eight cases, degenerative spondylolisthesis in nine cases, degenerative scoliosis in six cases, and postoperative revision in three cases. In addition, five patients were diagnosed with osteoporosis. The index levels included L2–3 in three patients, L3–4 in 13 patients, L4–5 in 23 patients, L2–4 in three patients, and L3–5 in six patients. The operation time was 81.1 ± 6.4 (range 65–102) min. Intraoperative blood loss was 39.9 ± 8.5 (range 15–72) mL. No severe complications occurred, such as nerve or blood vessel injuries. The patients were followed up for 11.7 ± 2.3 (range 4–18) months. At the last follow-up, the VAS decreased from 6.2 ± 2.3 to 1.7 ± 1.1, and the ODI decreased from 48.4% ± 11.2% to 10.9% ± 5.5%. Radiography showed satisfactory postoperative spine alignment. No cage displacement was found, but cage subsidence 2–3 mm was found in five patients without obvious symptoms, except transient low back pain in an obese patient. The lumbar lordosis recovered from 36.8° ± 7.9° to 47.7° ± 6.8°, and intervertebral disc height recovered from 8.2 ± 2.0 mm to 11.4 ± 2.5 mm. The spondylolisthesis rate decreased from 19.9% ± 4.9% to 9.4% ± 3.2%. The difference between preoperative and last follow-up was statistically significant (*P*<0.05).

**Conclusion:**

SA-LLIF can provide immediate stability and good results for lumbar degenerative diseases with a standalone anchored cage without posterior internal fixation.

## Introduction

Lumbar spinal fusion is a common treatment for a range of severe lumbar degenerative diseases, including degenerative spinal canal stenosis, instability, spondylolisthesis, and scoliosis. According to different approaches, lumbar fusion surgery can be divided into posterior lumbar interbody fusion (PLIF), transforaminal lumbar interbody fusion (TLIF), anterior lumbar interbody fusion (ALIF) and LLIF. LLIF can be divided into direct/extreme lateral interbody fusion (D/XLIF) and OLIF [1]. PLIF involves laminectomy and decompression through the posterior approach, but the dural sac and nerve root need to be retracted for interbody fusion, which may result in severe injury. Another posterior transforaminal approach for fusion is TLIF, which can reduce the risk of nerve traction injuries. The minimally invasive approach TLIF (MIS-TLIF) further reduces tissue injury, but requires entering the spinal canal, which poses a risk for dural tear and nerve root injury and could damage the lower back muscles and destroy the posterior column structure. The pedicle internal fixation may cause adjacent segmental degeneration and low back pain [2, 3]. ALIF and LLIF can avoid lumbar muscle injury and posterior column destruction, completely remove the diseased intervertebral disc, and better restore disc height and physiological kyphosis. However, ALIF needs to distract the internal organs and separate the large ventral blood vessels, which is relatively complicated and has the possibility of sympathetic nerve injury and retrograde ejaculation [2].

Lateral lumbar interbody fusion (LLIF) has developed rapidly and received increasing attention in recent years. It is a lumbar interbody fusion technique that is performed through a small incision and extraperitoneal approach without low back muscle splitting. After the degenerative intervertebral disc is exposed and removed, instant stability is obtained when the cage is implanted laterally [4, 5]. Extreme lateral lumbar interbody fusion (XLIF) and oblique lateral lumbar interbody fusion (OLIF) are the two major approaches to LLIF [1]. As transpsoas XLIF may interfere with the lumbar plexus nerve, OLIF has gained popularity because it passes through the natural gap between the psoas muscle and the abdominal aorta, which can significantly reduce the risk of nerve injury and achieve indirect decompression and reduction [6]. With the popularity of OLIF, the displacement and subsidence of cages has gradually increased, especially in standalone cages without assisted posterior fixation or anterior titanium plate fixation [7–9]. Woods et al. [10] reported that the incidence of complications after OLIF was 11.7%, and that of endplate collapse, the most common complication, was 4.4%. Le et al. [11] reported that the incidence of cage displacement or subsidence after OLIF was 14.3% in 140 cases. Therefore, in recent years, many scholars have suggested that OLIF should be performed in combination with posterior pedicle fixation to improve stability [12, 13]. However, OLIF combined with posterior fixation significantly increases surgical procedures, operative time, and complications related to internal fixation [10]. Even with anterior titanium plate fixation, spinal exposure must be expanded to deal with the segmental vessels. The anterior plate occupies the psoas major muscle attachment point and squeezes the lumbar plexus nerve. Both anterior and posterior fixation weaken the advantages of minimally invasive surgery [14].

Given these, we use a self-anchored cage with anchoring plate to perform standalone LLIF surgery, that is, self-anchored lateral lumbar interbody fusion (SA-LLIF). An oblique incision from the projection midpoint of the target disc onto the abdominal skin was used in our study, which can reduce the distraction of the abdominal wall when the cage is implanted. As the cage was implanted laterally via natural space, this procedure can also be called the lateral approach. This technique increases the stability of the cage by anchoring the plate and avoids posterior or anterior titanium plate fixation. Moreover, zero-profile plates were inserted into the cage and the adjacent vertebral body to avoid excessive exposure during the surgery. The self-anchored cage would not affect the anatomical position of the psoas major muscle and lumbar plexus nerve, so that minimally invasive surgery could eventually be achieved. The aims of this study were to: (1) determine the operation points of SA-LLIF, (2) evaluate the clinical and radiographic outcomes of patients who underwent SA-LLIF, and (3) analyze the indications for SA-LLIF.

## Materials and methods

### Population

Patients with lumbar degenerative diseases treated with SA-LLIF between January 2019 and June 2020 at Tianjin Hospital were reviewed. This study was approved by the Ethics Committee of Tianjin Hospital (2022 − 188). All patients underwent static (anteroposterior [AP] and lateral) and dynamic (flexion-extension) radiographs, computed tomography (CT), magnetic resonance imaging (MRI), and bone mineral density examination. The inclusion criteria for surgery were the following: (1) low back and leg pain were aggravated during standing activities or changes in position, and symptoms relieved by more than half while lying; (2) the diagnosis was consistent with one of the following: degenerative lumbar instability, degenerative lumbar spondylolisthesis grades I-II (Meyerding grade), lumbar disc herniation with endplate inflammation, low back pain > leg pain, lumbar scoliosis instability and mechanical low back pain, degenerative instability of adjacent or same segment after lumbar spine surgery; (3) Oswestry Disability Index (ODI) > 30%; (4) failure of conservative treatment for more than 3 months. The exclusion criteria included the following: (1) combined with psychosis or other cognitive disorders, which can affect the evaluation; (2) pathological fracture, tumor, or infection diseases; (3) lumbar spondylolisthesis (Meyerding grade) > grade II; (4) simple lumbar disc herniation, lumbar spinal stenosis with claudication-like pain and developmental spinal stenosis; (5) severe facet joint hyperplasia; (6) severe osteoporosis.

### Surgical technique

**Fig. 1:**
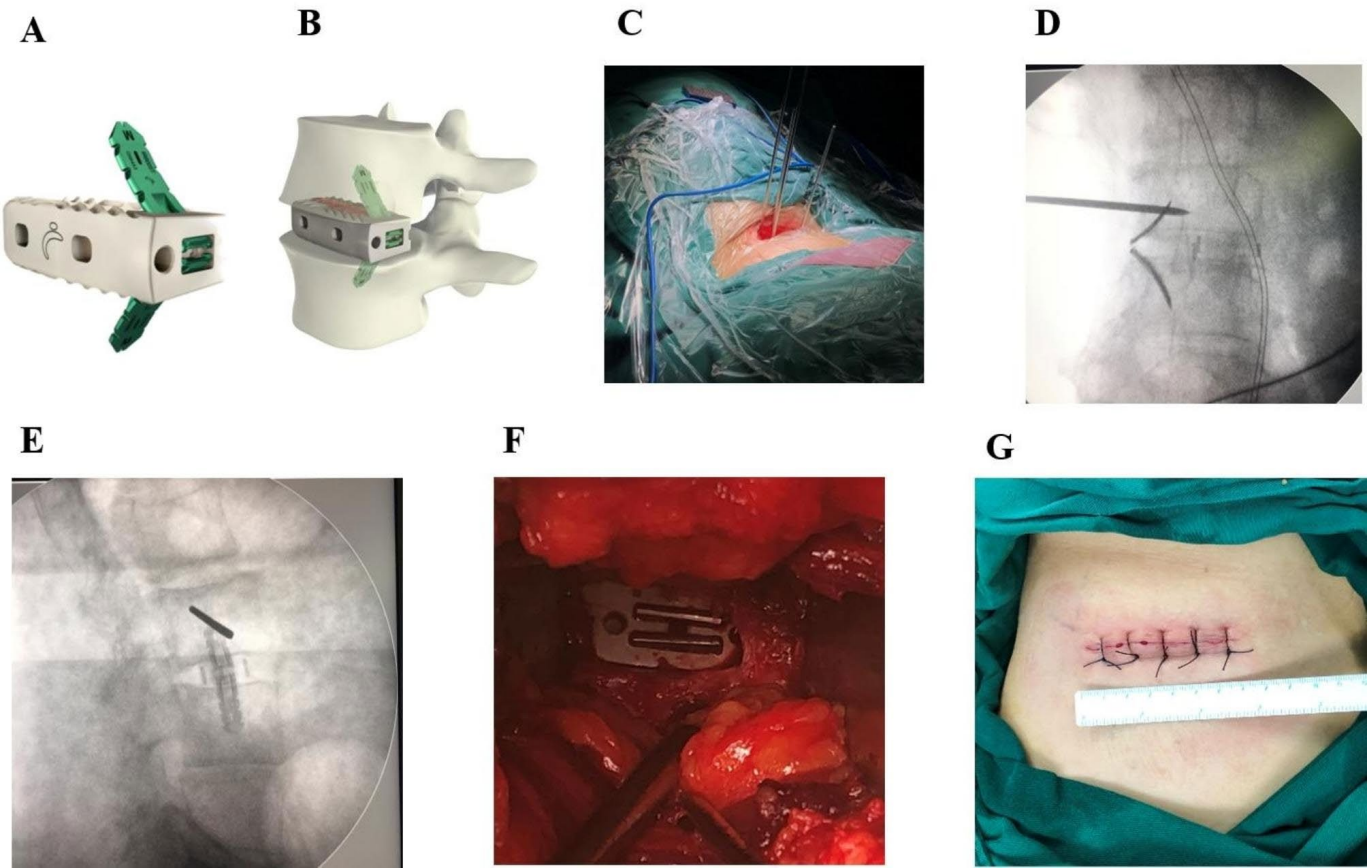
Schematic diagram of zero-profile cage with anchoring plates and intraoperative findings. (**A**) Cage with anchoring plates; (**B**) The cage filled with autologous bone was implanted into the intervertebral space, and the plates were inserted into the adjacent vertebrae; (**C**) The standard lateral decubitus position and incision; (**D, E**) AP and lateral radiographs showed that the cage was located in the middle of the disc space and the plates anchored the adjacent vertebrae; (**F**) The plates were submerged into the cage under the direct view and the cage was zero-profile; (**G**) The surgical incision was about 6 cm

General anesthesia was routinely administered in standard right lateral decubitus position and left approach. The hips and knees were slightly flexed to relax the psoas muscle. An oblique incision of approximately 6 cm was made from the midpoint of the target disc space to the ventral side. The abdominal muscles were bluntly dissected along the muscle fibers and pulled back and upward. To expose the intervertebral disc, two right-angled retractors were used to distract the psoas major muscle on the dorsal side and the extraperitoneal fat on the ventral side. The intervertebral disc was removed and endplate preparation were carefully performed. The disc space was then distracted by the spreader. Generally, the distraction height did not exceed the mean value of the adjacent normal disc space or was 2–4 mm more than the preoperative height. Combined with AP and lateral fluoroscopy, a trial spacer of appropriate size and lordosis angle was selected to moderately distract the disc space. An appropriate self-anchored cage was selected based on the size of the trial spacer. The anterior superior iliac bone was exposed through the same incision, and the autogenous iliac cancellous bone was scraped and packed into a self-anchored cage (Avenue-L, Zimmer Company, Fig. 1).

After installing the cage on the controller and adjusting the depth blocker, the position of the cage was confirmed via lateral and AP imaging. A medium anchoring plate was routinely selected for the single-level operation; in the two-level operation, a small plate was selected for the middle vertebral body and a medium-sized plate was used for the distal and proximal vertebral bodies (to avoid collision between the two plates). First, the distal vertebral body was anchored. The plate was covered on the mounting rod, slid into the slot of the cage, and tapped into the distal vertebral body, which was monitored by AP radiography. Similarly, another plate was inserted into the slot of the cage and proximal vertebral body, and the plate was completely embedded in the cage under direct vision (Fig. 1F). The two levels were operated in the same manner in the two spaces. Bleeding was stopped and the wound was irrigated before suturing by layers. No wound drainage was performed in any of the patients.

### Postoperative management

One day after surgery, the patients ambulated with braces or a wide waistband, and AP and lateral radiographs were taken. A brace or wide waistband was used until 3 months after the operation, to avoid weight bending and twisting; More strenuous physical activity is not allowed until at least 3 months after surgery. Nonsteroidal anti-inflammatory drugs were not used unnecessarily.

### Clinical and radiographic evaluation

Operative time and intraoperative blood loss were recorded, as well as complications including neurological injury, vascular injury, psoas major muscle weakness, thigh numbness, infection of the surgical site, and reoperation. Visual analog scale (VAS) and ODI scores were evaluated preoperatively, at the 3-month follow-up, and at the last follow-up [15]. Cage subsidence and spinal alignment were evaluated using lumbar radiography. Subsidence was defined as cage invading the vertebral endplate more than 2 mm on radiography. The subsidence was presented on postoperative radiographs, which was defined as early (intraoperative) collapse or subsidence. No subsidence was observed on postoperative radiography; however, during follow-up, subsidence occurred, which was defined as late collapse or subsidence. X-rays and CT scanning were used to evaluate fusion. Fusion was defined as bridging bone observed between vertebral bodies and implants, less than 5◦ of angular motion, less than or equal to 3 mm of translation, and an absence of radiolucent lines around more than 50% of either of the implant surfaces [7]. Lumbar lordosis (L: the angle between the L1 superior endplate and the S1 superior endplate [11]), intervertebral disc height (H: the average value of the anterior and posterior edge height of the disc space [13]), and the rate of spondylolisthesis (S: the ratio of the distance of spondylolisthesis to the upper endplate of the adjacent distal vertebral body [16]) were measured using lateral radiography.

### Statistical analysis

The data were processed and handled using SPSS (version 17.0; SPSS Company, Chicago, IL, USA) and are presented as mean ± standard deviation ($\bar x \pm S$). A paired *t*-test was used to compare the variable values pre- and postoperatively. Statistical significance was set at *P* < 0.05.

## Results

**Fig. 2:**
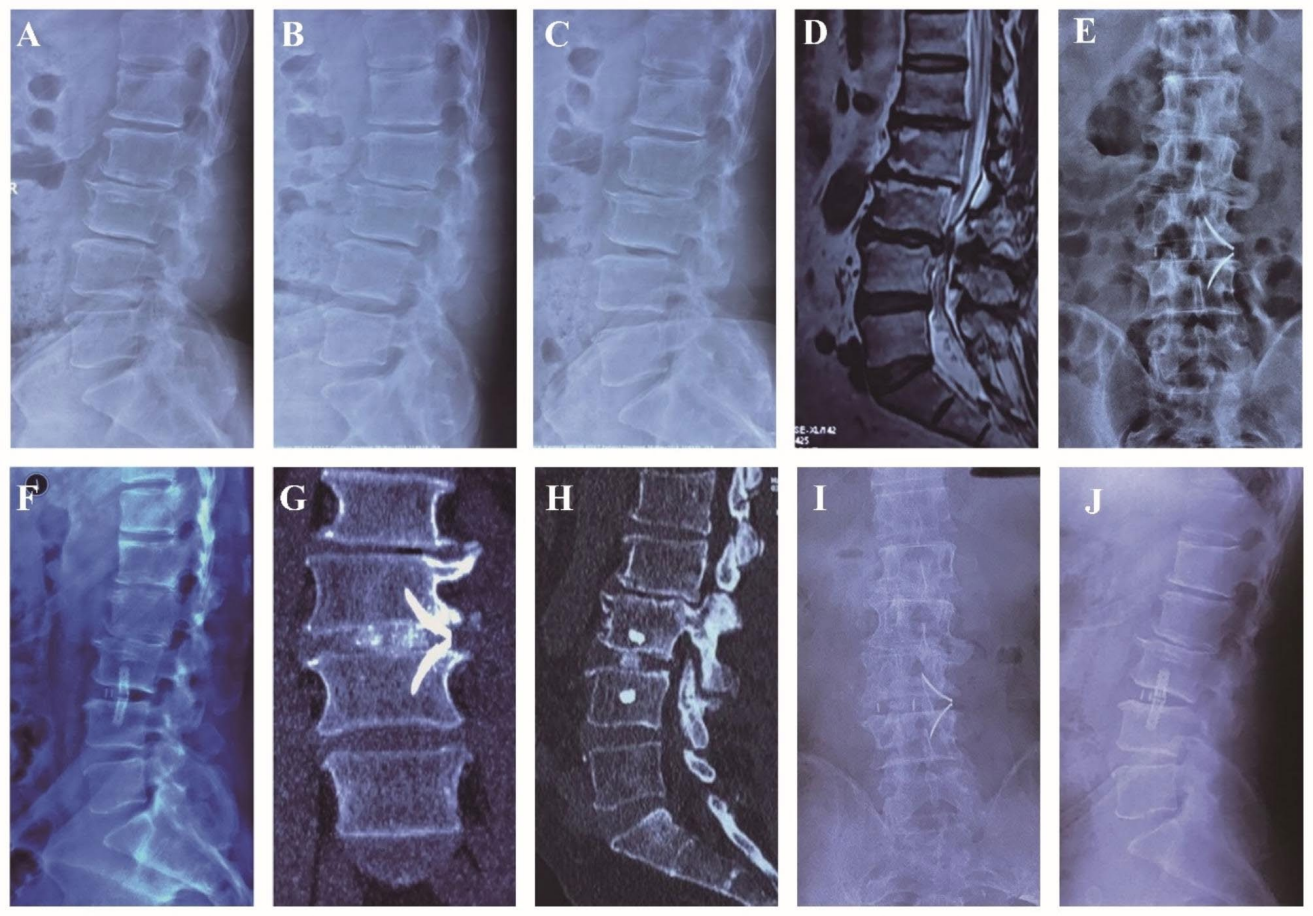
Pre-, Post-operative and follow-up images obtained in a 70-year-old male who had low back pain that exacerbated during activity, and was diagnosed with L3-4 degenerative spondylolisthesis. The symptoms disappeared after SA-LLIF. (**A**) The lateral X-ray shows lumbar spondylolisthesis of grade I; (**B**) The flexion X-ray shows reduction of spondylolisthesis; (**C**) The extension X-ray shows exacerbation of spondylolisthesis; (**D**) MRI shows L3-4 intervertebral disc degeneration and herniation; (**E F**) postoperative AP and lateral radiographs shows that cage was located in the center of the intervertebral space and good sagittal alignment was achieved; (**G, H**) CT coronal/sagittal position shows good position of cage, plate and bone graft; (**I, J**) 3 months after operation, the AP and lateral X-ray shows the plate and cage were stable

**Fig. 3:**
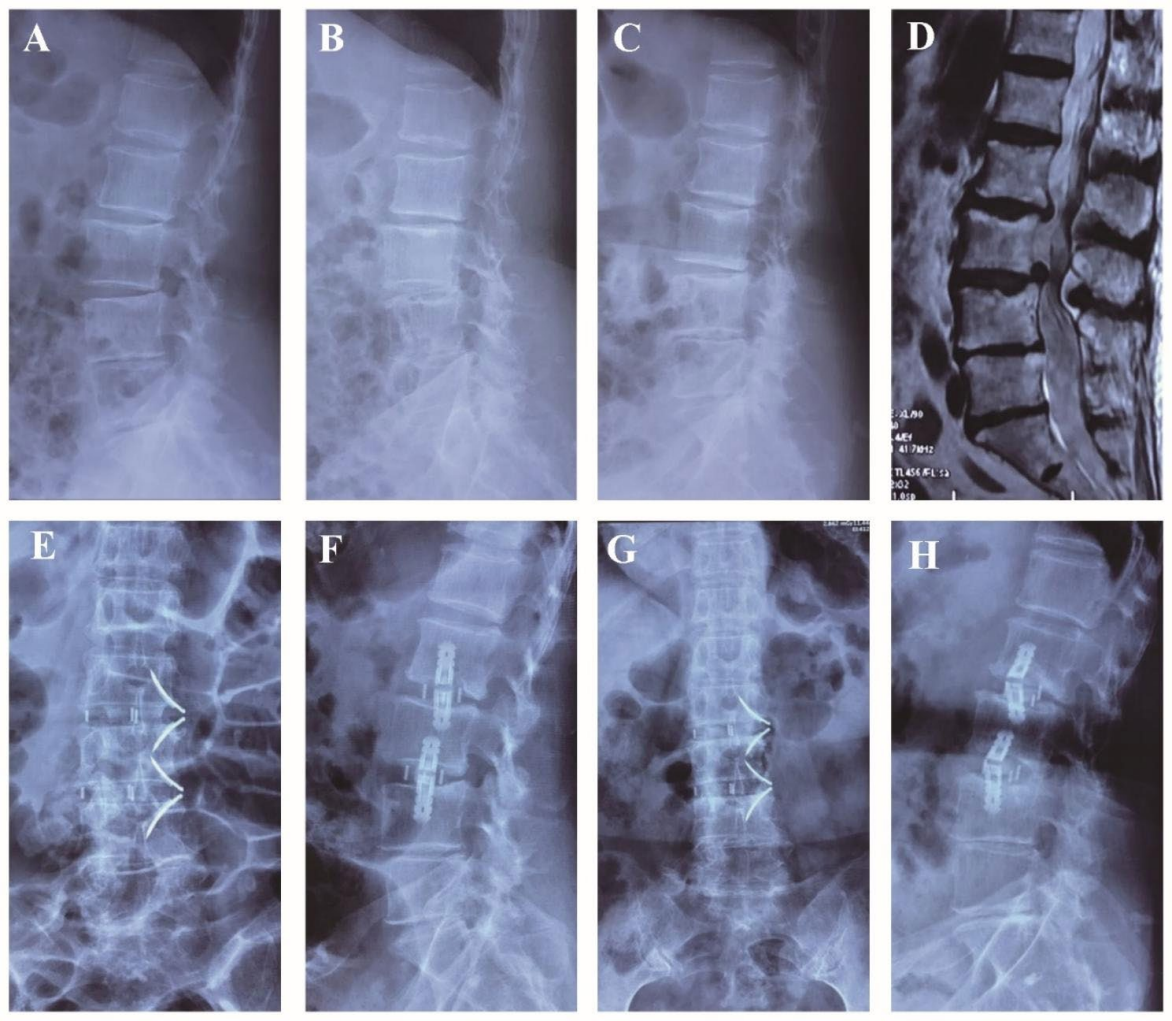
Pre-, Post-operative and follow-up images obtained in a 76-year-old female who had low back pain with radiating pain in the lower extremity, exacerbated during exercise and presented with L2-4 instability. The symptoms disappeared after SA-LLIF. (A) The lateral X-ray shows lumbar degenerated spondylolisthesis; (B, C) The flexion-extension lateral X-rays shows L2-4 instability; (D) MRI fat-suppressed image shows L3-5 intervertebral disc degeneration and herniation; (E, F) good lumbar alignment and satisfactory fixation were achieved after operation; (G, H) 3 months after operation, the AP and lateral X-ray shows the plate and cage were stable

**Fig. 4:**
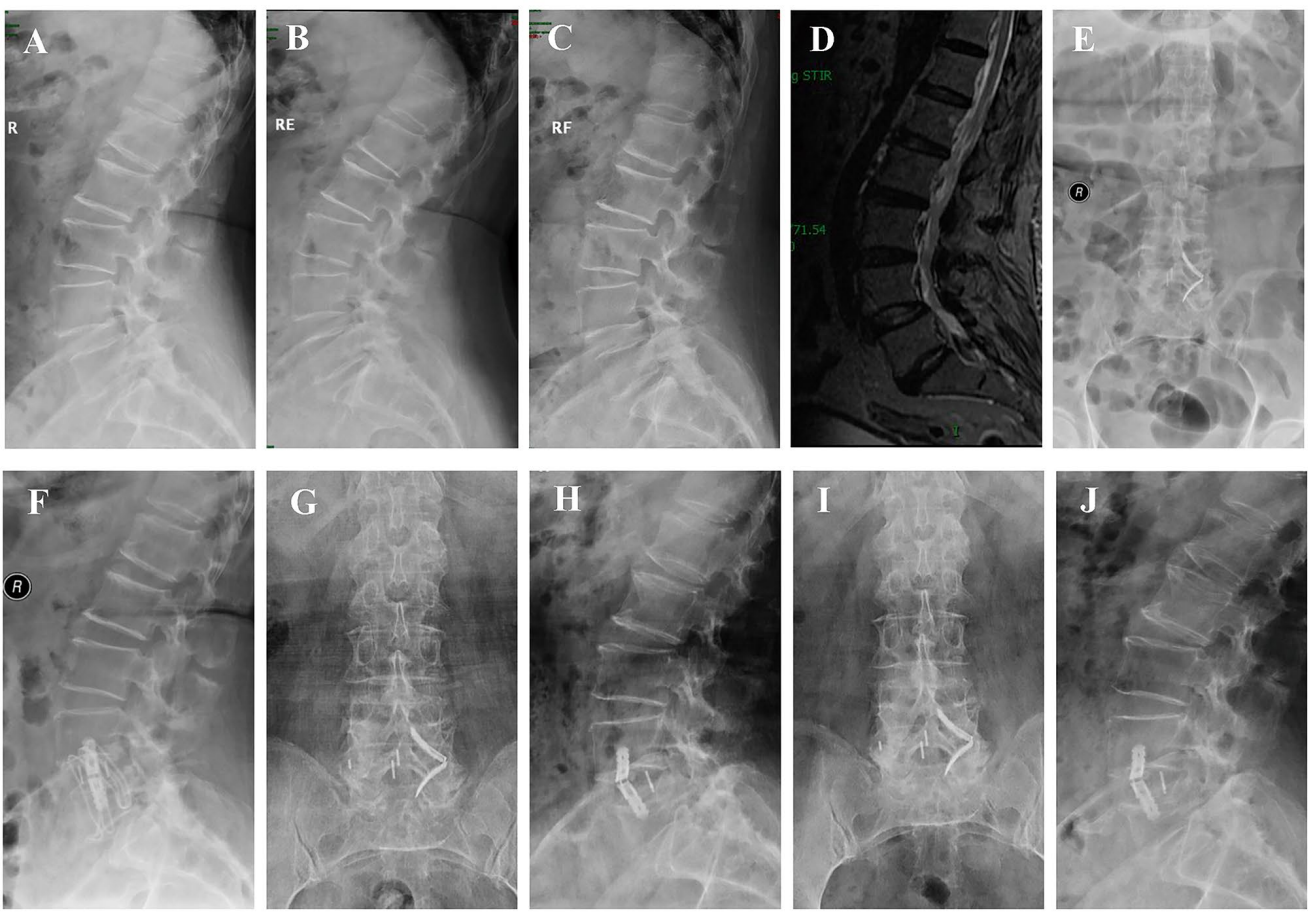
Pre-, Post-operative and follow-up images obtained in a 71-year-old female who had low back pain with radiating pain in the left lower extremity, exacerbated during exercise and presented with L4-5 degenerative spondylolisthesis. The symptoms were significantly improved after SA-LLIF. (**A**) The lateral X-ray showed lumbar degenerated spondylolisthesis; (**B, C**) The flexion-extension lateral X-rays showed L4-5 spondylolisthesis which exacerbated in the extension position; (**D**) MRI fat-suppressed image showed L4-5 intervertebral disc degenerated spondylolisthesis; (**E, F**) The postoperative AP and lateral X-rays shows recovery of L4-5 vertebral alignment; (**G, H**) 3 months after operation, the lateral X-ray shows that the upper endplate of the distal vertebral body collapses 2 mm, and the cage subsided. But the position of the plate and cage were stable; (**I, J**) 12 months after operation, the lateral X-ray shows the plate and cage were still stable

**Fig. 5:**
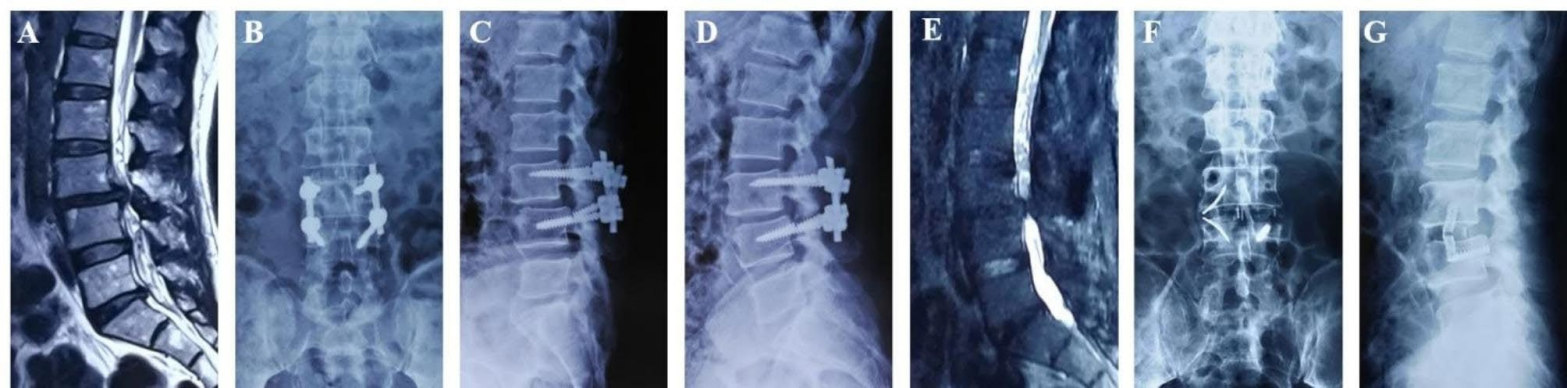
Pre- and Post-operative images obtained in a 61-year-old male treated with L3-4 posterior decompression and internal fixation 19 months before and diagnosed with internal fixation rupture and degeneration of the same level. The patient still had low back pain and lower extremity radiating pain after surgery and worsened in the past 1 month. The symptoms were significantly relieved after one-stage posterior internal fixation removed and right approach SA-LLIF. (**A**) preoperative T2-weighted MR images showed L3-4 degeneration and instability; (**B**) the AP X-ray showed that the pedicle screw on the left side of L4 broke; (**C, D**) the lateral X-rays of flexion-extension showed L3-4 instability; (**E**) preoperative sagittal T2-weighted MR showed L3-4 disc degeneration and protrusion; (**F, G**) the AP and lateral radiograph after revision showed that the left broken screw of L4 remained, and the intervertebral height and physiological arc of L3-4 recovered satisfactorily

The demographic data of the patients are shown in Table 1. There were 22 patients with lumbar stenosis and instability, eight with disc degenerative disease, nine with lumbar degenerative spondylolisthesis (Meyerding grade I), six with lumbar degenerative scoliosis, two with adjacent segment instability with spinal canal stenosis after lumbar internal fixation (including one with internal fixation loosening after posterior revision surgery), and one with screw fracture with the same segment spondylolisthesis after lumbar internal fixation. All the patients successfully completed the surgery (Typical cases were shown in Figs. 2, 3, 4 and 5). Five patients had osteoporosis, and one patient was obese (BMI 32.8) with decreased bone mass. Of these 48 patients, 39 underwent single-level surgery and nine underwent two-level surgery (three at L2–3, 13 at L3–4, 23 at L4–5, three at L2–L4 and six patients at L3–L5). The operation time was 81.1 ± 6.4 (range 65–102) min, and the intraoperative blood loss was 39.9 ± 8.5 (range 15–72) mL.

**Table 1:** Patient Demographic Data (n = 48)

**Characteristic**	
Years (range)	61.4 ± 7.3(range 49 ~ 78) years
Sex (M/F)	20/28
Follow-up after surgery, mean, months	11.7 ± 2.3 (range 4 ~ 18) months
**Diagnosis, n**	48
lumbar stenosis and instability	22
disc degenerative disease	8
degenerative spondylolisthesis	9
degenerative scoliosis	6
postoperative revision	3
**Fused Levels**	
single-level	39
L2-L3	3
L3-L4	13
L4-L5	23
two levels	9
L2-L4	3
L3-L5	6

### Pain score

As shown in Tables 2, 48 patients had a VAS score of 6.2 ± 2.3 preoperatively, 1.9 ± 1.1 at the 3-month follow-up, and 1.7 ± 1.1 at the last follow-up, which revealed a statistically significant difference (*P* < 0.05). Of 48 patients, the ODI score decreased from 48.4% ± 11.2% preoperatively to 14.2% ± 5.7% at the 3-month follow-up, and 10.9% ± 5.5% at the last follow-up (*F* = 328.69, *P* = 0.00). In comparison with the preoperative ODI, the difference was significant at the 3-month follow-up and the last follow-up (Table 3).

**Table 2:** Comparison of preoperative and postoperative VAS score of different groups($\bar x \pm s$)

Pathogenesis	n	Pre op.	3 m Follow-up	Last Follow-up	*F*	*P*
Lumbar stenosis and instability	22	6.4 ± 2.1	1.8 ± 1.0^*^	1.6 ± 1.0^*^	74.72	0.00
Disc degenerative disease	8	6.0 ± 2.9	2.1 ± 1.1^*^	1.9 ± 1.2^*^	11.56	0.00
Degenerative spondylolisthesis	9	5.9 ± 2.4	1.7 ± 1.2^*^	1.6 ± 1.1^*^	19.64	0.00
Degenerative scoliosis	6	5.5 ± 2.4	1.7 ± 1.0^*^	1.7 ± 1.2^*^	10.46	0.00
Postoperative revision	3	6.7 ± 2.5	2.7 ± 1.5	2.3 ± 1.5	4.76	0.06
Total	48	6.2 ± 2.3	1.9 ± 1.1^*^	1.7 ± 1.1^*^	120.56	0.00

The numerical expression with * was statistically different from the preoperative comparison. (P<0.05)

*VAS, visual analogue scale.*

**Table 3:** Comparison of preoperative and postoperative ODI score of different groups ($\bar x \pm s$,%)

Pathogenesis	n	Pre op.	3 m Follow-up	Last Follow-up	*F*	*P*
Lumbar stenosis and instability	22	49.2 ± 9.8	14.8 ± 5.8^*^	10.7 ± 5.2^*^	188.618	0.00
Disc degenerative disease	8	47.9 ± 16.0	13.1 ± 6.2^*^	11.6 ± 5.4 ^*^	31.441	0.00
Degenerative spondylolisthesis	9	47.2 ± 11.0	12.6 ± 5.6 ^*^	10.0 ± 6.7 ^*^	58.958	0.00
Degenerative scoliosis	6	48.2 ± 11.3	15.3 ± 5.2^*^	12.3 ± 5.7 ^*^	38.26	0.00
Postoperative revision	3	47.3 ± 15.4	15.3 ± 8.4^*^	9.7 ± 7.0 ^*^	10.43	0.01
Total	48	48.4 ± 11.2	14.2 ± 5.7 ^*^	10.9 ± 5.5 ^*^	328.69	0.00

The numerical expression with * was statistically different from the preoperative comparison. (P<0.05)

*ODI, Oswestry Disability Index.*

### Radiographic evaluation

Radiographs showed that the spinal alignment was significantly improved after surgery and follow-up, and there was no lateral or AP displacement or dislocation of the cage. Three patients had early (intraoperative) vertebral endplate collapse of 1–1.5 mm. The collapse was slightly aggravated at the 3-month follow-up after the operation, and cage subsidence reached 1.5 mm in one patient and 2.5 mm in two patients, without obvious clinical symptoms. Thirteen patients had a late vertebral endplate collapse of 1–3 mm at 3 months postoperatively, of whom 5 patients had cage subsidence of 2–3 mm. All of them were asymptomatic except for one obese patient with temporary low back pain, and there was no obvious aggravation of endplate collapse and subsidence at the last follow-up. Fusion was observed in 89.5% of the operative levels. What’s more, neither pseudoarthrosis nor movement were observed in these unfused levels.

The lumbar lordosis of 48 patients at the 3-month follow-up (48.4 ± 7.1°) and the last follow-up (47.7 ± 6.8°) was significantly larger than that before operation (36.8 ± 7.9°) (*P* < 0.05) (Table 4). The disc height of the operative level at the 3-month follow-up and the last follow-up (11.4 ± 2.5 mm) also demonstrated significant improvement compared with that before the operation (8.2 ± 2.0 mm) (*P* < 0.05, Table 4). The rate of spondylolisthesis in nine patients significantly decreased from 19.9 ± 4.9 at pre-operation to 9.4 ± 3.2 at the last follow-up (*P* < 0.05) (Table 4).

**Table 4:** Date of preoperative and postoperative Radiological Examination ($\bar x \pm s$)

Indicators	n	Pre op.	3 m Follow-up	Last Follow-up	*F*	*P*
Lumbar lordosis(°)	48	36.8 ± 7.9	48.4 ± 7.1 ^*^	47.7 ± 6.8^*^	38.24	0.00
disc height(mm)	48	8.2 ± 2.0	11.5 ± 2.7 ^*^	11.4 ± 2.5 ^*^	29.77	0.00
Slippage rate(%)	9	19.9 ± 4.9	9.2 ± 5.1 ^*^	9.4 ± 3.2 ^*^	16.70	0.00

The numerical expression with * was statistically different from the preoperative comparison. (P<0.05)

### Complications

Eight patients complained of pain in the area of the iliac bone harvested, which was improved via conservative therapy; three patients presented numbness in the distal inguinal region of the incision and anterior medial thigh, which was relieved through symptomatic treatment; three patients had a swollen abdomen on the operative side, which was improved through banding treatment; and there was one case of a slightly weaker hip flexion on the operative side compared to that on the contralateral side, which recovered spontaneously 1 week after the operation. One obese patient experienced low back pain when he increased activity 2 weeks after the operation. The radiograph showed that the cage subsided by approximately 3 mm, and the symptoms improved after reducing activity and taking non-steroidal drugs. There were no reported incision and surgical site infection, acute nerve injury, vascular rupture, or organ damage.

## Discussion

LLIF via the lateral approach was reported by Mayer et al. [4] in 1997, which not only avoids posterior structural disruption and complicated anterior separation but also reduces related complications. However, when DLIF or XLIF passes through the psoas major muscle, there is a risk of damage to the lumbar plexus nerve in the psoas major muscle, which can cause weakness of the iliopsoas and quadriceps femoris, thigh pain, or numbness with an incidence of 20% [14]. In 2012, Silvestre et al. [5] used the natural space between the psoas major muscle and great blood vessels to access the intervertebral disc, which reduced the risk of lumbar plexus injury. This technique is called OLIF. LLIF or OLIF distracts the abdominal muscles via a small incision to avoid muscle damage. The diseased intervertebral disc was fully removed to achieve indirect decompression by stretching the disc space. As LLIF or OLIF does not enter the spinal canal, it could reduce the risk of nerve injury, epidural adhesion, and bleeding. LLIF and OLIF have the advantages of a small incision, fewer complications, and rapid recovery; thus, these techniques have been widely applied [17].

However, with the increasing application of LLIF or OLIF, many studies have reported that the rate of cage displacement and subsidence is higher, especially in standalone LLIF or OLIF without assisted posterior fixation [8, 9]. According to Abe et al. [13], the overall complication rate was 48.3% in standalone OLIF, and the incidence of endplate fracture and cage subsidence was 18.7%. In our study, nearly half of the patients who underwent standalone OLIF had endplate collapse or cage subsidence of varying grades, which may be related to the match between the vertebral body and the fusion cage interface. Patients with cage subsidence less than 2 mm generally had no reported symptoms and needed continuous observation. However, for those with significant cage subsidence (> 2 mm), the stability and indirect decompression effects would be influenced. Patients with clinical symptoms often require posterior surgical revision and pedicle internal fixation to improve stability. In addition, lateral displacement or even prolapse of the cage after OLIF is often reported, which is related to the lack of stability. Consequently, many scholars currently recommend LLIF or OLIF combined with posterior fixation to increase stability, especially in patients with osteoporosis or endplate injury. However, the lateral approach combined with posterior fixation significantly increases the complexity of the surgical procedure, operative time, trauma, and cost. Compared with MIS-TLIF, the advantages of LLIF or OLIF are compromised.

SA-LLIF, with the use of a cage with an anchoring plate, was performed to solve the insufficient stability of the standalone LLIF or OLIF. A biomechanical study showed that the SA-LLIF anchoring plate could achieve immediate stability, and the range of motion after anchoring was smaller than that of the control group, which indicated that the stability exceeded that of the normal specimens [16]. The fixation and loading interface were increased by the anchoring plate, which not only prevented cage displacement but also eliminated micro-movement in all directions. It has been reported that micromovement requires less than 28 μm during bony fusion, and fibrous tissue healing is likely to occur when micromovement is greater than 150 μm, which would result in pseudoarthrosis. Moreover, the plate inserted into the vertebral body increases the stress area against the axial load, which could share the stress of the endplate and eventually reduce the incidence of cage subsidence eventually [18, 19]. In addition, the plate penetrates the bony endplate to insert into the vertebral body, causing the bone marrow blood in the cancellous bone of the vertebral body to infiltrate the cage, which could promote bony fusion. The anchoring plate is completely submerged into the cage (zero-profile), which does not affect the anatomical location of the psoas major muscle and the lumbar plexus nerve.

Forty-eight patients in our study (including five patients with osteoporosis and one obese patient) were treated with SA-LLIF without posterior fixation, which greatly simplified the surgical procedure and reduced trauma. The VAS and ODI scores significantly decreased after surgery and follow-up. Spinal alignment was significantly improved after surgery and during follow-up in the study, and there was no obvious displacement or dislocation of the cage and plates. Lumbar lordosis and disc space height significantly increased at the follow-up. Compared with the spondylolisthesis rate before surgery, the spondylolisthesis rate after surgery improved by 61.4%. This may be related to loosening of the disc space and distraction reduction. At the last follow-up, there was a high fusion rate in this study. In addition, there was no significant difference in the degree of correction loss during the follow-up, indicating that SA-LLIF can provide appropriate stability. Cage subsidence is influenced by various factors, including bone mineral density, endplate strength, cage material and type, as well as surgeon’s manipulation (such as endplate injury, placement position, and intervertebral space distraction height). According to Le [11]et al. research, cage subsidence can be categorized into radiographical subsidence and clinical subsidence. Radiographical subsidence refers to any postoperative X-ray evidence of endplate fracture; whereas clinical subsidence includes radiological subsidence along with symptoms such as recurrent pain, neurological symptoms, and deterioration of clinical efficacy associated with indirect decompression reduction. Knox [20]et al. further classified cage subsidence into mild (less than 2 mm), moderate (3–5 mm), and severe (greater than 6 mm) categories. In our study, of the thirteen cases of postoperative subsidence observed, only five cases exhibited moderate subsidence without any accompanying clinical symptoms, indicating that the disc space was relatively stable. This proportion was lower compared to the reported rate of 2.1% for clinical subsidence in Le [11]et al. study and the rate of severe subsidence (32%) reported by Okano [21]et al. In this study, the higher incidence of cage subsidence may be attributed to the absence of posterior screw fixation and self-anchoring on one side. Additionally, elderly patients with reduced bone mineral density were found to have a higher risk for cage subsidence [22]; therefore, the rate of cage subsidence in this study also may be related to the older age.

An obese patient developed low back pain when activity increased two weeks after the operation. The X-ray showed that the cage subsided by approximately 3 mm, which was considered to be related to the body weight, large amount of activity, and low bone mass. However, the cage subsided in parallel with no obvious displacement or dislocation, and plate fixation was stable. Therefore, symptoms improved after conservative treatment. Eight patients do have iliac crest pain after the surgery. In fact, in this study, the harvest was mainly cancellous bone obtained from the small rectangular bone window opened on the upper surface of the anterior superior spine via the same surgical incision. It’s less invasive and the incidence of pain is relatively low. The symptoms of these patients were all relieved after conservative treatment. The numbness in the inguinal area and anterior medial thigh was related to the interference of the cutaneous nerve caused by incision in three cases, which improved after symptomatic treatment. Three patients presented with an abdominal eminence on the operative side, which may be related to paralysis of the abdominal wall muscles. The patient’s symptoms improved after conservative treatment. One patient had a transient decrease in hip flexion muscle strength, which may be related to the traction of the psoas muscle and lumbar plexus.

SA-LLIF achieved self-stabilized zero-profile fusion using an anchoring cage with plates inserted into the vertebral body. Because the vertebral body is a cancellous bone, its internal fixation may not be as strong as pedicle screw fixation. However, biomechanical experiments and clinical applications have suggested that it can provide good initial stability and effectively avoid posterior internal fixation. All patients in this group had lumbar degenerative diseases, including lumbar instability, degenerative lumbar spinal stenosis with instability, grade I-II degenerative spondylolisthesis, lumbar disc herniation with endplate inflammation with more serious lower back pain than leg pain, lumbar scoliosis with instability and degeneration, and instability of adjacent segments or the same segment after lumbar surgery. These lesions mainly involve intervertebral discs. In addition, good results were obtained by removing the diseased intervertebral discs and restoring the spinal alignment intervertebral sequence and stability. No significant differences were observed between these lesions. A probable explanation for this is that the above diseases can all be used as indications for SA-LLIF. However, SA-LLIF does not result in sufficient reduction in patients with lumbar spondylolisthesis greater than grade II. It is also difficult to provide an exact indirect decompression effect in patients with severe lumbar spinal stenosis using SA-LLIF. In patients with severe facet joint hyperplasia and a stiff intervertebral space, the lesions are mainly concentrated on the posterior side, making it difficult to perform SA-LLIF. Therefore, these symptoms should not be used as indications. Although five cases of osteoporosis and one case of obesity in this group also achieved good results, whether this is a routine indication remains to be further studied. Patients with severe osteoporosis and vertebral endplate fractures, which make it difficult to support the cage, should be treated with caution. The short-term effect on the 48 patients in this group was good. It can be argued that SA-LLIF alone can provide good stability and avoid posterior fixation. This significantly simplifies the LLIF procedure when appropriate indications are selected.

The present study has some limitations. First, it was a retrospective study. Randomized controlled clinical trials need to be conducted in future studies. Second, the follow-up period was relatively short. Finally, more patients should be included in the study to further demonstrate its effects and values. Therefore, a prospective trial with a larger sample with long-term follow-up is necessary.

In conclusion, good stability and great simplification in surgical procedures were observed in SA-LLIF without assisted posterior screw fixation, when appropriate indications were selected. In addition, the anchored plate is completely submerged into the cage with a zero profile, which does not affect the anatomical location of the psoas major muscle and lumbar plexus nerve. Above all, non-instrumented standalone SA-LLIF does not compromise clinical outcomes in some patients.

## Data Availability

The datasets used and/or analyzed during the current study are available from the corresponding author on reasonable request.

## Abbreviations

LLIF: lateral lumbar interbody fusion
OLIF: oblique lateral lumbar interbody fusion
MIS-TLIF: minimally invasive transforaminal lumbar interbody fusion
AP: anteroposterior
CT: computed tomography
MRI: magnetic resonance imaging
VAS: visual analogue scale
ODI: Oswestry disability index
